# Efficiency of All-Trans Retinoic Acid on Gastric Cancer: A Narrative Literature Review

**DOI:** 10.3390/ijms19113388

**Published:** 2018-10-29

**Authors:** Damien Bouriez, Julie Giraud, Caroline Gronnier, Christine Varon

**Affiliations:** 1 INSERM, U1053, Bordeaux Research in Translational Oncology, 33000 Bordeaux, France; damienbouriezbt@gmail.com (D.B.); julie.giraud@u-bordeaux.fr (J.G.); caroline.gronnier@chu-bordeaux.fr (C.G.); 2 Department of Digestive Surgery, Haut-Lévêque Hospital, 33000 Bordeaux, France; 3 Department of Life and Health Sciences, University of Bordeaux, 33000 Bordeaux, France

**Keywords:** tretinoin, retinoic acid, stomach neoplasms, cancer stem cell, CD44, retinoic acid receptor, retinoic X receptor, differentiation therapy

## Abstract

Gastric cancer (GC) is the third leading cause of cancer-related death worldwide with a five-year survival rate of around 25%, and 4% when diagnosed at a metastatic stage. Cancer stem cells (CSC) have recently been characterized as being responsible for resistance to radio/chemotherapies and metastasis formation, opening up perspectives for new targeted therapies. Those CSCs express biomarkers such as cluster of differentiation 44 (CD44) and display high aldehyde dehydrogenase activity that converts vitamin A-derived retinal into retinoic acids. All-trans retinoic acid (ATRA), which has pro-differentiating properties, has revolutionized the prognosis of acute promyelotic leukemia by increasing its remission rate from 15% to 85%. Recent studies have started to show that ATRA also has an anti-tumoral role on solid cancers such as GC. The purpose of this review is therefore to summarize the work that evaluated the effects of ATRA in GC and to evaluate whether its anti-cancerous action involves gastric CSCs targeting. It has been demonstrated that ATRA can block the cell cycle, enhance apoptosis, and decrease gastric CSCs properties in GC cell lines, tumorspheres, and patient-derived xenograft mice models. Therefore, retinoids and new synthetic retinoids seem to be a promising step forward in targeted therapy of gastric CSC in combination with existing chemotherapies. Future studies should probably focus on these points.

## 1. Introduction

Gastric cancer (GC) is currently the third leading cause of cancer-related death and the fifth most frequent cancer worldwide [1]. It has a poor prognosis with a five-year relative survival around 25% in Europe [2], and 4% when metastatic. This poor prognosis may be explained by diagnosis at an advanced stage, as well as resistance to conventional chemotherapies, with a high rate of recurrence as distant and peritoneal metastasis. Interestingly, GC incidence decreases by about 1.5% per year because of a better control of its risk factors, the main one being infection by the bacteria *Helicobacter pylori* [3]. Its eradication indeed halves the risk of GC. Other GC risk factors are autoimmune atrophic gastritis, and to a lesser degree, heredity, ethnicity, alcohol, and tobacco consumption, infection with Epstein Barr virus, and a history of partial gastrectomy [4]. Lauren’s histological classification distinguishes intestinal type gastric adenocarcinoma from the diffuse type [5]. Non-epithelial GCs, which are less common, are mainly represented by mucosa associated lymphoid tissue (MALT) lymphomas, followed by endocrine tumors and, exceptionally, stromal tumors. This review will focus mainly on gastric adenocarcinomas.

GC treatment consists of either surgery associated with perioperative chemotherapy, or palliative chemotherapy when diagnosed at the metastatic stage [6,7]. Interestingly, a subpopulation of cancer cells has been identified as resistant to chemotherapy and responsible for a high recurrence rate, explaining the poor prognosis of this disease [8]. Those cells are the so-called cancer stem cells (CSCs), which were identified as cells involved in tumor initiation and propagation. They are characterized by self-renewal and asymmetrical division properties, being at the origin of the more or less differentiated cells composing the tumor mass [8]. Ongoing research thus focuses on the development of more potent therapies to target those CSCs. Gastric CSCs (GCSCs) were essentially characterized by the expression of the cell-surface protein cluster of differentiation 44 (CD44), and by high aldehyde dehydrogenase (ALDH) activity [8,9,10,11,12,13]. Among the ALDH enzymes, the ALDH 1A1 and ALDH 1A3 isoforms display a retinaldehyde dehydrogenase (R-ALDH) activity, which converts vitamin A-derived retinal into active retinoic acids (RAs).

**Figure 1:**
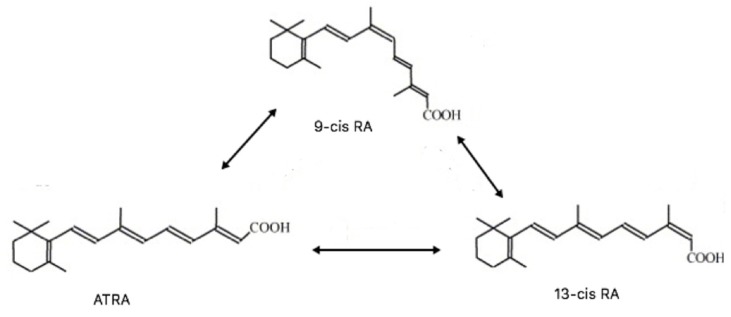
Retinoic Acids (RA) stereoisomers.

RAs include three isomers such as all-trans retinoic acid (ATRA), 9cis retinoic acid (9cisRA), and 13cis retinoic acid (13cisRA) (Figure 1) [14].

Retinoids are ligands of retinoic acid receptors (RARs) and retinoic X receptors (RXRs), which act mainly as RAR–RXR heterodimers and are intranuclear ligand-dependent transcription factors for proteins implicated in cellular differentiation [15].

In acute promyelotic leukemia (APL), a reciprocal translocation is acquired on the *RAR* gene on chromosome 17 and, in the majority of cases, on the promyelotic leukemia (*PML*) gene located on chromosome 15. This translocation leads to the synthesis of a PML-RARα fusion protein that induces a promyelotic stage blockage and leukemia initiation [16].

Nowadays, ATRA is considered as a current treatment for APL [17] and has increased its remission rate from 15% to 85% [18].

In this model, ATRA allows PML-RARα fusion protein to initiate expression of genes implicated in granulocyte differentiation by specifically targeting RARα. APL patients are also treated with arsenic, which targets PML protein [15,17,18].

The antiproliferative and differentiation properties of ATRA have also been shown in GC cells-xenotransplanted mice [19] demonstrating that it can display its activity even in tumoral cases without PML-RARα translocation.

In addition, other in vivo studies, have demonstrated that vitamin A, ATRA, 13cisRA, or recently developed synthetic retinoids can suppress growth of several other types of xenotransplanted or chemically induced tumors (mammary cancer [20], lymphoma [21], head and neck cancer [22], and melanoma [23]).

This article reviews literature about the antitumoral effect of ATRA and focuses on its specific activity against GCSCs in order to better understand the way they could be targeted to improve GC prognosis.

This review will successively describe (i) GCSCs and their biomarkers, (ii) the history of ATRA as an anticancer drug and its targets, and finally (iii) the effect of ATRA on GC models.

## 2. GCSC and Biomarkers

In response to *H. pylori* infection, gastric stem cells that have the longest lifetime are susceptible to acquire genetic or epigenetic modifications that can lead to CSC formation. Another hypothesis suggests that GCSCs can also originate from bone marrow-derived stem cells that homes into the gastric mucosa in response to *H. pylori* infection and contributes to metaplasia and dysplasia [12,24,25].

**Figure 2:**
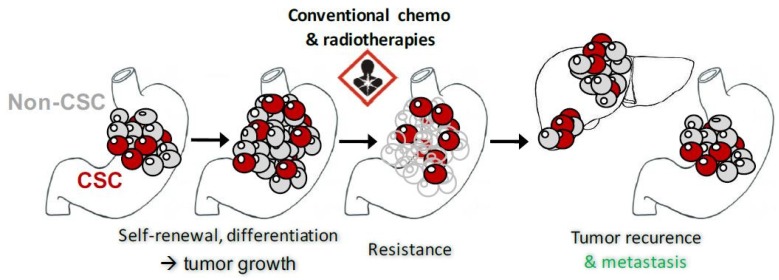
Schematic representation of gastric cancer stem cells (GCSC) properties.

It has recently been demonstrated that CSCs, which represent a small percentage of the cancer cells, are at the origin of the more or less differentiated cells in the tumor mass. Also, CSCs have particular properties compared to non-CSCs cancer cells. Their most important property is their capacity to make asymmetrical divisions in order to generate a new CSC and a more differentiated non-CSC progenitor. These differentiation properties allow CSCs to reconstitute the tumor heterogeneity observed within the primary tumor [8,26]. CSCs are able to form tumorspheres in tridimensional culture conditions in vitro and initiate tumor growth when injected in low cell numbers in immunocompromised mice. CSCs have shown increased resistance to conventional chemotherapy, for example in colorectal cancer [27,28], and radiotherapy for example in glioblastoma [29] (Figure 2).

As CSCs are more resistant to treatments than the more differentiated cancer cells, they may lead to cancer recurrence after treatment. They are therefore a potential target in cancer therapy to avoid metastasis, recurrence, and radio-chemotherapy resistances [8,26]. Their chemoresistance was shown to be associated with the expression of drug efflux systems, such as ATP binding cassette transporter proteins, as well as a high ALDH activity [30,31,32]. In vitro studies demonstrated that cancer cells expressing CD44 and ALDH initiate more tumorspheres than CD44− and ALDH− cancer cells [11]. Xenograft experiments using extreme limiting dilution analysis mathematical models demonstrated that CD44+ and ALDH+ cells initiate more tumors in vivo than cells expressing other biomarkers such as CD133 [11].

CD44 is expressed, at a very low level, in the isthmus of healthy gastric glands where stem cells reside. It was reported that these CD44+ stem/progenitor cells expand from the isthmus towards the base of the unit in metaplastic and dysplastic areas induced in response to chronic *H. pylori* infection [12,13,33]. This migration occurs via epithelial to mesenchymal transition (EMT), a process in which epithelial cells acquire migratory properties of mesenchymal cells conferring CSC-like properties to CD44+ cells [13,25,34]. CD44+ cells also display other characteristic features of CSCs such as invasion [35,36], endothelial cell adhesion [37], and drug resistance [38]. A recent study demonstrated that CD44 inhibition by peptide inhibitors prevents the development of cellular hyperproliferation and chronic atrophic gastritis in animal models of *H. pylori*–induced gastric carcinogenesis [39]. However, as CD44 is expressed in many different cancer cell types, it is not enough specific to be a putative target for GC therapy [40]. More potent CSC biomarkers were therefore screened in order to target more specifically GCSCs.

Interestingly, in GC, ALDH+ cells represent 1.6% to 15.4% of the tumor CSC and contain higher frequency of tumorigenic CSCs than CD44+ cells. ALDH activity was recorded mainly in CD44+ cells, showing that ALDH+ cells represented a subpopulation within the CD44+ subpopulation of cancer cells [11]. Nguyen PH et al. demonstrated that ALDH+ cells did not incorporate the nuclear dye Hoechst-33342 comparing to the ALDH− cells [11]. Treatment with drug efflux inhibitors such as Verapamil or Reserpin, restored the nuclear dye Hoechst-33342 incorporation in ALDH+ cells and sensitized them to conventional chemotherapy in vitro. Therefore, according to Nguyen et al., ALDH+ cells seem to be more resistant to conventional chemotherapeutic drugs than ALDH− cells thanks to their drug efflux ability.

ALDH activity could thus be a putative biomarker GC chemoresistance.

Unfortunately, the Aldefluor assay, used to detect CSC in vitro through ALDH activity, cannot be used to sense CSCs in tissue biopsies.

ALDH has many isoforms, which are expressed by 19 different genes [41]. Several studies have shown that some ALDH isoforms allow the detection of CSC on tumor tissue, but the isoform is different depending on the type of cancer and from one tumor to another. Among them, the main isoforms expressed in tumors are R-ALDH, ALDH1A1, and ALDH1A3 (to a lesser extent, ALDH3A1) responsible for the oxidation of retinaldehyde to RA [41]. They can metabolize and detoxify chemotherapeutic agents, such as cyclophosphamide in hematopoietic stem cells, and their high level of expression was shown to be predictive of poor therapeutic response in breast cancer [31]. Some ALDH isoforms seem, therefore, to be biomarkers of resistant CSCs.

These findings imply that ALDH activity and CD44 could be considered as putative targets to inhibit tumor growth and to overcome resistance to cancer therapy [11]. Treatments targeting these markers were therefore sought to improve GC prognosis.

## 3. ATRA Historic and Targets

The first connection between vitamin A and cancer was established as early as 1926, by Fujimaky et al., when it was found that GC appeared in rats fed with a vitamin A-deficient diet [42]. Vitamin A could not be administrated alone as an anticancer drug because high doses of this molecule were toxic to animals and man and induced hypervitaminosis A syndrome of which main symptoms are: bone pain, dizziness, headache, hypercalcemia, and skin and hair changes. Therefore, a search for less toxic and more potent analogs of vitamin A was undertaken. The first compound that was investigated intensively was ATRA [42].

The first in vitro study was achieved by Breitman et al. in 1980 who demonstrated that butyrate, dimethyl sulfoxide, and RA induced differentiation of the HL-60 leukemia cell line [43]. The Shanghai Institute of Hematology therefore decided to conduct the first clinical study with 24 APL patients who were given ATRA alone; of these, 23 cases achieved complete response with differentiation of promyelocytes [44].

In APL, ATRA was used rather than its isomer 13cisRA because it showed better anticancer efficiency in vitro and in vivo [45]. Therefore, ATRA is nowadays considered as a current treatment for APL [17].

In addition, retinoids are known to inhibit carcinogenesis because they inhibit growth, induce differentiation, and cause cell death in many other types of cancer cells (e.g., mammary gland cancer, neuroblastoma, and GC) [19,20,21,22,23]. They inhibit carcinogenesis by interacting with RARs and RXRs, which are intranuclear ligand-controlled transcription factors [46]. RAR and RXR are members of the steroid and thyroid hormone receptor superfamily. Each family contains three receptors (α, β, and γ), encoded by different genes. RARβ’s and RARγ’s protein sequence presents, respectively, 100% and 91% coverage with RARα’s protein sequence [47].

ATRA is solubilized, protected against oxidation-reduction reactions, and transported into the nucleus by cellular retinoic acid binding proteins (CRABP). Its biological effects are then mediated in the nucleus by RAR and RXR, which act as heterodimers to bind specific DNA promoting sequences, so called the retinoic acid response elements (RARE), of target genes [15]. 

In the absence of ligand, RAR and RXR recruit co-repressors, such as the nuclear co-repressor (N-Cor) and the silencing mediator of RA, and thyroid hormone receptor (SMRT), which in turn recruit histone deacetylases proteins. This multiprotein complex repressor induces chromatin condensation and thus downregulates target genes expression.

**Figure 3:**
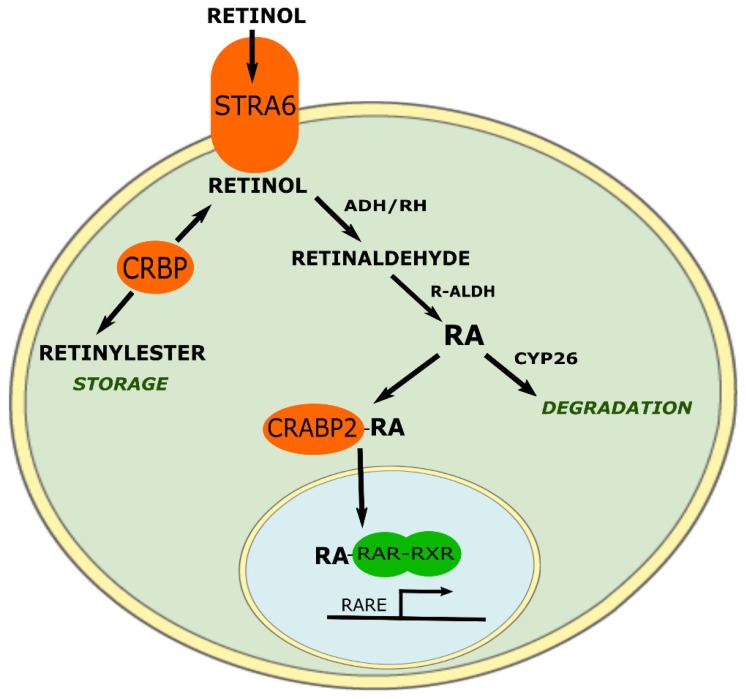
Schematic representation of RA’s signaling via the RAR-RXR pathway. ADH: aldehyde dehydrogenase, CYP26: cytochrome P450 26A1, CRPB: cellular retinol binding protein, CRABP2: cellular retinoic acid binding protein 2, RARE: retinoic acid response element, STRA6: stimulated by retinoic acid 6.

On the other hand, in presence of ATRA, CRABP proteins facilitate its interaction with RAR or RXR, act as a coactivator [48] and recruit proteins which have either a histone acetyltransferases activity, such as SRC/p160, GCN5/pCAF, or CBP/P800, or a methyl transferase activity such as CARM1. These multiprotein complexes induce chromatin decondensation thus targeting gene expression [49] (Figure 3).

### 3.1. Retinoic Acid Receptor α (RARα)

It has been demonstrated that ATRA is a RARα ligand, and that a specific antagonist of RARα can counteract ATRA’s effect [50,51] (Table 1).

RARα is expressed in myeloid stem cells and allows, in physiological concentrations of RA (about 1.9 ng/mL), granulocyte differentiation [17,52]. In the example of APL, a reciprocal translocation is acquired on the *RAR* gene on chromosome 17, and, in most of the cases, on the *PML* gene located on chromosome 15 or more rarely on the promyelotic leukemia zinc finger (*PLZF*) gene located on chromosome 11 resulting in a PML-RARα or PLZF-RARα fusion protein. This PML-RARα fusion protein, which interacts strongly with corepressors, such as N-Cor and SMRT, is insensitive to physiological doses of RA and cannot display transcriptional activation of reporter genes thus blocking the differentiation of myeloid cells and resulting in the typical phenotype of leukemia.

In contrast, pharmacological doses (about 350 ng/mL) [52] of RA induces the dissociation of PML-RARα’s corepressors and activates its transcriptional activity, and thus myeloid cells differentiation. As corepressors interact even stronger with PLZF-RARα, these doses of ATRA do not dissociate it from corepressors and cannot activate its transcriptional activity for myeloid cells differentiation.

In conclusion, pharmacological doses of RA induce cell differentiation in APL patients bearing PML-RARα but not PLZF-RARα translocation.

The sensitivity of APL to ATRA was the first example of a therapeutic strategy based on cell differentiation.

Moreover, many studies show that retinoids can inhibit growth of precancerous lesions, such as cervix dysplasia [53] and leukoplakia [54], and of several solid tumors, such as mammary cancer [20], lymphoma [21], head and neck cancer [22], melanoma [23], and GC [19], demonstrating that RAs have a differentiation property even in cases without PML-RARα translocation, which is restricted to APL. In those cases, ATRA activates RARα (or other RARs) and regulates signaling pathways to carry out its anticancer function.

In the example of breast cancer, treatment with ATRA caused a dose-dependent decrease in the abundance of Pin1, which is an isomerase coordinating multiple phosphorylation events during oncogenesis. ATRA therefore inhibits its substrate oncoproteins including cyclin D1, HER2, ER-α, AKT, NF-κB, p65, c-Jun, and PKM2, and increases tumor suppressors activity such as SMAD2, SMAD3, and SMRT [55].

Two studies demonstrated that WNT10B and WNT3, proto-oncogene glycoproteins of the WNT family, which are implicated in embryonic development and carcinogenesis through activation of WNT-beta-catenin pathway, are downregulated in the NT2 pluripotent human embryonal carcinoma cell line after ATRA treatment [56,57].

RA also seems to activate tumor suppressive human inductible nitric oxyde synthase (hiNOS) pathway, which significantly inhibits tumor formation [58].

In conclusion, ATRA seems to display its anti-cancer action, at least partly, through RARα via different signaling pathways including Pin1, WNTs and hiNOS.

Furthermore, it has been demonstrated that RA can also enhance apoptosis and inhibit proliferation in lung [59] and breast cancer lines, through RARβ induction [60].

### 3.2. Retinoic Acid Receptor β (RARβ)

Many studies show that ATRA induces RARβ’s expression at both protein and mRNA levels [61,62], proving that ATRA might mediate its action not only by RARα, but also by RARβ. Moreover, according to Liu G et al., a loss of RARβ’s expression parallels ATRA resistance in esophageal squamous carcinoma EC109 cell line [61].

To our knowledge, no study about the effect of the interaction between ATRA and RARγ has yet been conducted.

### 3.3. Retinoic X Receptors (RXR)

RXR acts, with RAR, as a heterodimer and is also a nuclear ligand-dependent transcription factor. It was therefore considered as a potential target of RA and studies were conducted to better understand whether RA signaling could pass through this latter.

It was shown in 1990 that ATRA could not bind RXRs. Another retinoid isomer was suspected to bind it [63], and indeed, in 1992, 9cisRA was found to bind RXRs and RARs [64] (Table 1).

In conclusion, ATRA can bind RARs but not RXRs; thus, after ATRA treatment, its isomerization in 9cisRA is necessary to obtain a signal through RXR.

### 3.4. RAR and RXR’s Expression Impact on GC Prognosis

As RAR and RXR are targets of RAs, it has been investigated whether these proteins can be used as prognosis markers or markers of therapeutic response.

In healthy human tissue, RARα and RXRs are ubiquitously expressed, while RARβ and RARγ display a more restricted distribution pattern, with RARγ being predominantly expressed in the skin [77].

According to Hu KW et al., RARα, RARβ, RARγ and RXRγ are expressed in significantly lower levels in GC tissue sections, with lower RARβ, RARγ, and RXRα expression significantly related to advanced stages and lower levels of RARα and RARβ in tumors with poor histopathologic grade. Moreover, a low expression of RARα independently predicts an unfavorable prognosis in GC and the overall survival of ATRA treated patients is significantly longer for RARα positive than RARα negative GC patients [78].

A low expression of *RARβ* has also been elucidated in many other cancers than GC such as thyroid cancer [79], epithelial carcinoma, head and neck cancer [80], prostate cancer [81], esophageal cancer [82], and lung cancer [83].

It has also been shown that high expression of RARβ is correlated with favorable patient prognosis. Furthermore, a better response to RA has been demonstrated when RARβ is overexpressed through transfection in human neuroblastoma cell lines [72].

However, in a contradictory way, RARα was an independent indicator of poor prognosis in oral squamous cell carcinoma [84] and in breast cancer [85].

After identifying ATRA’s targets and its mechanism of action, this review investigates whether studies evaluated its action on the GC example.

In conclusion, RARβ seems to be a marker of favorable prognosis and therapeutic response. Whereas, due to controversial studies, RARα is not sufficient on its own to be a robust prognosis marker.

## 4. ATRA on GC Models

As early as in 1926, Fujimaky et al., found that GC appeared in rats fed with a vitamin A-deficient diet [86]. Then, in 1985, epidemiological studies demonstrated that vitamin A plasmatic concentration was lower in patients with gastric dysplasia suggesting that RA could have an impact on gastric carcinogenesis [87].

To further understand the effect of ATRA on GC, studies have been conducted and three main anti-cancerous effect of this molecule have been identified: (i) inhibition of cell cycle and induction of cell differentiation [88,89,90,91,92]; (ii) pro-apoptotic action [88,93,94,95], and (iii) inhibition of CSCs properties [88,96,97] (Table 2).

GC cell lines seem to be a relevant model since RARs seem to be expressed in a large variety of them [61,98].

### 4.1. ATRA’s Mechanism of Action for Cell Cycle Blocking and Differentiation Initiation

It has been demonstrated that ATRA and 9cisRA display their growth inhibition properties through p21WAF1/CIP1 induction, which decreases the expression of cyclin dependent kinase (CDK) 4 and 2, leading to the arrest of cell cycle progression [89]. ATRA can inhibit AP1, which is a transcription factor implicated in inflammation and cell proliferation [91] through RARα and RARβ in GC cell lines [90]. ATRA has shown a decreasing effect on GC cell line growth by downregulating the ERK/MAPK pathway, which is implicated in cellular proliferation, survival, differentiation, migration, and angiogenesis [92]. Moreover, according to Nguyen et al., ATRA induces the expression of GCSCs differentiation markers such as cytokeratin 7 (KRT7), osteopontin (SSP1), cytokeratins (PanCK), mucin 6 (MUC6), and trefoil factor 3 (TTF3) in tumorspheres from two GC cell lines and in mice with subcutaneous tumor xenografts of two GC cell lines and two patient derived xenografts (PDX) [19].

According to Li T et al., a combination of ATRA, Sorafenib, and miRNA in encapsulated nanoparticles inhibit GC cells proliferation and initiate apoptosis in vitro [88].

### 4.2. ATRA’s Mechanism of Action for Apoptosis Initiation

It has been shown that ATRA can mediate its anticancer activity by enhancing apoptosis in breast cancer cell lines, which highly express the orphan receptor TR3, and cell cycle blockage when TR3 is less expressed [93]. TR3 is also responsible for apoptosis in GC cancer cells when translocated into the mitochondria in response to 9cisRA activation of RXRα [94].

It was also demonstrated that ATRA induces apoptosis markers, such as PDCD4, and cleaved caspase 3 in GC cell lines tumorspheres and in PDX models [19].

Furthermore, it was recently demonstrated that the association of γ-secretase inhibitors to ATRA increases its growth inhibition and apoptosis enhancement properties [95].

### 4.3. ATRA’s Mechanism of Action for CSC Properties Inhibition

Tumorspheres and mice subcutaneous PDX initiation have been defined as the main properties of CSCs [8,9,26]. According to Nguyen et al., ATRA inhibits tumorsphere formation and survival in a dose-dependent manner [19]. It has been demonstrated that ATRA reduces tumor growth in GC PDXs or GC cell lines xenograft models [88,96]. Moreover, it has been shown that there are less mice bearing liver metastasis, after intrasplenic xenograft of GC cells (BGC 823 and MKN45), when they were fed with ATRA rather than with a control treatment (33–50%) [97]. According to Li T et al., a combination of ATRA, Sorafenib, and miRNA in encapsulated nanoparticles also seem to have an antitumor effect in subcutaneous GC cell lines xenografted mice [88]. Moreover, it was demonstrated that ATRA downregulates the expression of CSC markers (CD44 and ALDH) and stemness genes, such as KLF4 and SOX2, and inhibits GC PDX growth in immunocompromised mice [19].

**Figure 4:**
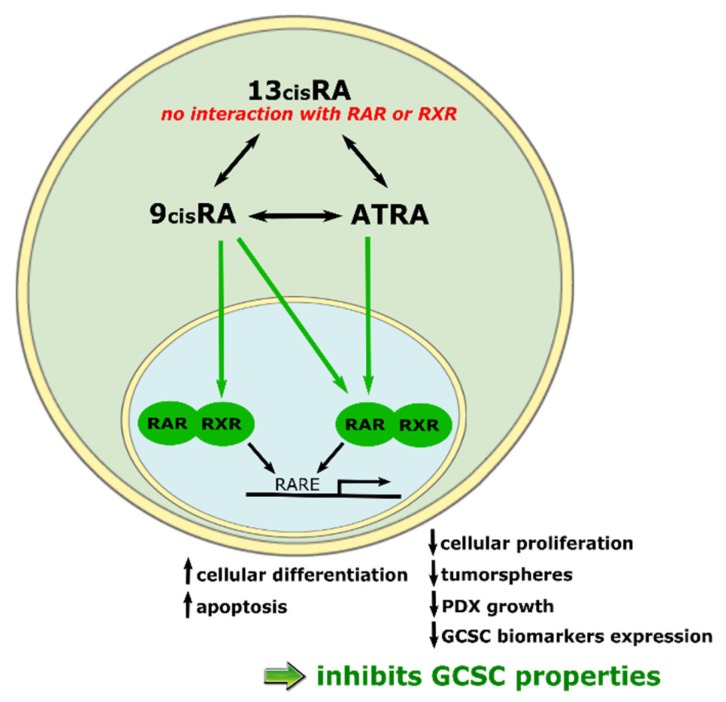
Schematic representation of ATRA’s signaling pathway and anticancer effects. double black arrows: possible isomerization between these two molecules; green arrows: isomer’s ability to bind RAR or RXR receptor; simple black arrow: RAR or RXR’s ability to bind RARE.

In conclusion, ATRA displays its anticancer action on GC by inhibiting cellular proliferation, inducing differentiation and apoptosis, inhibiting CSC properties such as tumorspheres formation and PDX growth in mice (Figure 4) (Table 2).

Studies evaluating the anti-cancer effect of ATRA on patients with GC were therefore screened.

### 4.4. ATRA’s Anticancer Effect on Patients with GC

Only two studies tested ATRA on patients with GC.

In 2015, Jin J et al. showed that patients with gastric dysplasia treated with omeprazole and sucralfate, associated to ATRA, presented a better attenuation of their dysplasia (68% vs. 37%) compared to patient treated with omeprazole and sucralfate alone. This was accompanied with an increased expression of the tumor suppressor *pRb* and a decreased expression of *HER2* oncogenic receptor in patient’s gastric mucosa [99].

Then, Hu K W et al., showed, on a cohort of 80 patients with GC, that ATRA could significantly prolong overall survival when combined with conventional chemotherapy. Also, the expression of RARα was correlated with the responsiveness to ATRA [78].

In this study, ATRA was added (or not) to conventional chemotherapies highlighting the possible synergic anticancer effect of ATRA with these treatments. The hypothesis of a synergy between these two treatments could be explained by a targeting of CSCs by ATRA and the rest of the tumor mass by cytotoxic chemotherapy.

### 4.5. ATRA’s Anticancer Effect on Non-Epithelial GCs

MALT lymphoma, which represents over 50% of primary gastric non-Hodgkin lymphomas, [100] are associated with H. pylori infection in 80 to 90% cases [101]. Its treatment, based on the eradication of H. pylori, provides 70 to 100% remission rates in localized disease [102]. As ATRA displays an anti-cancer activity on APL and some lymphomas and sarcomas [21], questions were asked about the effect of this molecule on non-epithelial GCs.

To our knowledge, no studies evaluated the effect of ATRA or other retinoids on MALT lymphomas, or gastric endocrine or stromal tumors.

### 4.6. Association of RA and Conventional Chemotherapies

The synergistic effect of ATRA and conventional chemotherapies has been demonstrated in different types of cancer and in different in vitro and in vivo models. ATRA showed a synergistic anticancer effect in combination with cisplatin on squamous carcinoma cell lines [103] PDX [104] and patients [105], on cervical carcinoma cell lines [106], and on lung metastasis formation in a mousse melanoma model [107]. Also, ATRA with 5fluoro-uracil chemotherapy has shown a synergistic anticancer effect on squamous carcinoma cell lines [103].

To further understand the mechanisms underlying this synergistic anticancer activity of ATRA and conventional chemotherapies, studies were conducted and demonstrated that: (i) ATRA inhibits cell growth and DNA synthesis [103,108]; (ii) ATRA pretreatment counteracts cisplatin resistance, and (iii) this combination reduces the fraction of CSCs and tumor dissemination [109].

Furthermore, it was demonstrated that ATRA displays a synergic anti-CSC effect, by facilitating apoptosis and cell cycle arrest, in combination with cisplatin or 5-fluorouracil on AGS GC and Kyse-30 esophageal squamous cell carcinoma cell line [110].

It has also been shown that RA interacts in vitro with conventional cytotoxic drugs, such as cisplatin and taxanes (paclitaxel and docetaxel), to decrease apoptosis threshold and block cell cycle [22].

In conclusion, RA appears to have a synergistic anticancer action with conventional chemotherapies, such as cisplatin or 5fluoro-uracil, on squamous cell carcinoma, non-small cell lung cancer, pancreatic adenocarcinoma, cervical carcinoma, metastatic melanoma, and gastro-esophageal carcinoma.

ATRA could reduce the high GC chemoresistance through the synergistic anticancer activity it displays with conventional chemotherapies. However, a resistance to RA has been described in APL patients [111,112].

A better understanding of mechanisms involved in the resistance to ATRA is therefore needed in order to find ways to avoid it and later treat patients bearing GC with this molecule.

### 4.7. Mechanisms of Resistance to ATRA

One of the principal mechanism of RA resistance is the induction of cytochrome oxidases, which are implicated in RA metabolism [113].

Consequently, inhibitors of cytochrome P450 (CYP450), such as Liarozole, can increase a patient’s ATRA plasmatic concentrations [114]. Nevertheless, cytochrome P450 26A1 (CYP26), a member of CYP450 superfamily, is now characterized as the main enzyme responsible for RA clearance. Indeed, R116010 is a CYP26 inhibitor and has a 100-fold increased potency compared to that of Liarozole in human T47D breast cancer cells. Also, as R116010 is more specific to RA metabolism, it shows less side effects than Liarozole [115].

Another study suggests that the activation of the orphan nuclear receptor PXR can increase ATRA metabolism, which might be a mechanism for some forms of ATRA resistance [74].

On the other hand, some studies suggest that 4-hydroxy RA, 18-hydroxy RA, 4-oxo RA, and 5,6-epoxy RA, four metabolites of ATRA, can activate RAR and RXR. For example, cells that are the most sensitive to ATRA are the ones that metabolize ATRA the most [116]. In addition, it has recently been shown that several ATRA metabolites exhibit significant biological activity in some cell lines [70,117,118].

Conversely, other cell lines, that are fast metabolizers of RA, are relatively resistant to ATRA [119].

In conclusion, it is unclear whether inhibiting ATRA’s first phase of metabolism is a way to increase its activity.

Another resistance mechanism could be drug efflux via permeability-glycoprotein (P-GP), a multidrug resistance transporter. The P-GP inhibitor, verapamil, restores the ability of ATRA to induce differentiation in ATRA-resistant APL cells expressing multidrug-resistance gene transcripts [120].

Also, an increased expression of CRABPs that may sequester RA has also been linked to leukemia cells resistance in several studies [121].

Translational studies on myeloid leukemia cells and clinical studies on APL patients demonstrated that, point mutations in the ligand-binding domain (E domain) of RAR is, as well, another possible resistance mechanism [111,122].

In conclusion, ATRA resistance seems to be due, at least partly, to drug efflux systems, an increased expression of CRABPs and point mutation in RARE. Therefore, P-glycoprotein inhibitors such as verapamil might be promising therapies to be associated to ATRA to treat patients with GC and avoid its resistance. ATRA’s isomers and synthetic retinoids have also been tested on many cancers in order to find the molecule with the least resistance and the best anticancer activity.

### 4.8. Other Retinoids

ATRA 13cisRA and 9cisRA can isomerize into one another, but at equilibrium, ATRA is the dominant isomer, accounting for approximately 60–70% of total RAs [123] (Figure 1).

#### 4.8.1. 13cisRA

13cisRA has more favorable pharmacokinetic properties than ATRA and 9cisRA with a 3-fold higher maximum tolerated dose, facilitating higher peak plasma levels (5–10 mM), and a significantly longer half-life explaining favorable in vivo activity [113].

According to Jiang Sy et al., 13cisRA has a stronger effect than ATRA on the regression of subcutaneous tumor xenografts derived from GC cell line SC-M1. However, it caused higher toxicity such as bone fractures and weigh loss. Mice treated with implantable 15 mg pellets of 13cisRA died after thirty days of the experiment [96].

The most relevant anticancer activity of 13cisRA was demonstrated in 1985 in neuroblastoma. It was demonstrated that 13cisRA decreased expression of the *N-MYC* oncogene in neuroblastoma cells in vitro [124]. After these results, phase I [125] and phase III [126] clinical trials demonstrated the efficacy of 13cisRA against neuroblastoma in pediatric patients.

13cisRA has also shown efficacy in reducing incidence of skin cancer and xeroderma pigmentosum [127], and lesions in Kaposi’s sarcoma [128].

Squamous cell carcinoma of the head and neck responds well to 13cisRA in combination with interferon α-2a [129]. However, 13cisRA has less activity than ATRA in mammal tumors derived from human mammary carcinoma cells xenografted in athymic mice [130].

Biochemical studies demonstrate that 13cisRA has very low affinity for RARs in comparison with ATRA or 9cisRA [70].

Two hypotheses explain how 13cisRA can have clinical efficiency on neuroblastoma and acne with a very low affinity for RARs. The first hypothesis is that 13cisRA’s activity may be the result of its binding to some other unknown receptor. More probably, the second hypothesis is that 13cisRA is subject to a biotransformation to an isomer of RA (ATRA or 9cisRA) that binds and activates one of the known families of receptors [123]. 

In conclusion, even if 13cisRA is currently given to treat neuroblastoma, in vivo studies of its effect on GC showed too much toxicity in comparison to ATRA which explained why it has never been tested on GC patients.

#### 4.8.2. 9cisRA

9cisRA can be generated from both its carotenoid precursors and isomerization of ATRA [131].

It is currently given to treat severe and refractory hand eczema [132].

In GC cell lines it has been demonstrated that, through the activation of RXR, 9cisRA can potentiate the ligand-activated transcription factor peroxisome proliferator-activated receptor γ (PPARγ)’s activity, which contributes to the inhibition of cell growth and tissue invasion ability, and enhances apoptosis [133].

Other experimentation confirmed anti-proliferative properties in thyroid cancer cell lines [134].

The combination of an RXR agonist, such as 9cisRA and PPARγ agonists, could induce maximal inhibitory effects on tumor growth and apoptosis.

However, 9cisRA displayed severe toxicity with significant weight loss compared to 13cisRA in rats with neuroblastoma xenografts [135].

It was demonstrated that RXRα, activated by 9cisRA, is responsible for TR3 nucleocytoplasmic translocation, which promotes apoptosis in GC cells [94]. 

In conclusion, even if 9cisRA is currently given to treat severe hand eczema, in vivo studies of its effect on GC showed too much toxicity, which explains why it has never been tested on GC patients.

#### 4.8.3. Fenretinide (RII)

Fenretinide (RII) is a synthetic retinoid introduced in 1993 that has shown growth inhibition and apoptosis induction in neuroblastoma [136], lung non-small cancer [137], and malignant hematopoietic [138] cell lines.

Liu G et al., demonstrated that RII displays growth inhibition in a BLC82 lung adenocarcinoma cell line and a BGC 823 GC cell line. Among all the cell lines he tested, EC109 esophageal squamous carcinoma cell line was the only one resistant to RA and RII in terms of growth inhibition, which parallels its loss of RARβ2 expression. Exogenous RARβ2 expression restores RII induced growth inhibition, suggesting that RII acts, at least in part, via the RARβ2 receptor [61].

Other in vitro studies demonstrated that RII inhibits GC cell line growth by retaining them in G1 cell cycle phase by acting either on RXRα or RARγ [139]. 

According to Formelli et al., RII seems to be effective against human ovarian carcinoma xenograft in mice and potentiates cisplatin activity [140].

Clinically, this drug has been shown to be effective in preventing contralateral cancer in women who have had breast cancer [141].

RII has also shown efficacy in relapsed or refractory neuroblastoma in a phase I study [142].

RII’s toxicities are diminished dark vision [141,143], and musculoskeletal complaints [144].

Yet no studies evaluated the effect of RII on patients bearing GC.

#### 4.8.4. ATPR

Also, 4-amino-2-trifluoromethyl-phenyl retinate (ATPR) is a novel synthetic retinoid that inhibits subcutaneous GC xenografts growth in mice model. It also decreases expression of COX-2 and increases expression of RARβ in subcutaneous GC xenograft mice models [145]. Proteomic analysis on a SGC79-01 GC cell line treated by ATPR demonstrated that its antiproliferative activity might be mediated by inhibition of AKT’s phosphorylation which inhibits cyclinE/CDK2 through FOXO1A and P27Kip1 upregulation [146].

ATPR better inhibits proliferation and migration of breast cancer MDA-MB-231 cells than ATRA. Its mechanism of action was associated with the down regulation of MLCK’s expression and phosphorylation of MLC proteins involving p38-MAPK pathway which induces cellular proliferation [147].

ATPR inhibits cell growth and cell migration in lung adenocarcinoma cell lines [148], GC cell lines [149], and hepatocellular carcinoma cell lines [150].

Yet, no study evaluated ATPR’s toxicity in comparison to ATRA as well as the anticancer effect of ATPR on patients with GC.

#### 4.8.5. ATRA-Podophyllotoxin Conjugate

In Zhang et al.’s study, ATRA was conjugated to Podophyllotoxin, which is a naturally-occurring aryltetralin lignan and displays its anticancer activity by inhibiting microtubules assembly. This synthetic molecule demonstrated an antiproliferative and a pro-apoptotic activity on MKN45 and BG823 GC cell lines by decreasing ERK and AKT expression and increasing RARα and RARβ expression [151].

## 5. Implications and Future Directions

ATRA is a molecule that has been known for about thirty years and had important success in the treatment of APL as a pro-differentiation therapy. Recent studies proposed that ATRA displays its pro-differentiation activity by specifically targeting CSC, cells that are responsible for GC poor prognosis by initiating metastasis and resistance to radio and chemotherapies.

Indeed, in vitro studies demonstrated that ATRA blocks cell cycle and enhances apoptosis in GC cell lines and in vivo studies demonstrated that ATRA reduces tumor growth in GC subcutaneous PDX models.

Thus, more studies are required to evaluate RA’s impact on GC cell invasion and metastasis formation.

However, ATRA has numerous adverse effects and CSC can acquire resistance. New synthetic retinoids, such as RII and ATPR, seem to have better anticancer efficiency than ATRA but they have not been tested yet on patients.

A better knowledge of new synthetic retinoids and studies testing combinations of treatments with conventional or new chemotherapies are needed and should offer new therapeutic options to treat GC.

## Figures and Tables

**Table 1:** Literature report of the interactions of different RAs with their receptors (green: an interaction is highlighted, orange: no interaction is highlighted, red: a lack of interaction is highlighted).

	ATRA	13cisRA	9cisRA
RARα	[50], [65,66,67,68]	[64], [69], [70]	[71]
RARβ	[51], [62], [61], [72], [65], [66], [73]	[64], [69], [70]	[71]
RARγ	[65], [66]	[64], [69], [70]	[71]
RXRα	[63], [74], [65]	[74]	[64], [74], [65], [68], [71], [75]
RXRβ	[63], [65]		
RXRγ	[63], [74], [65]	[74]	[74], [65], [68], [76]

**Table 2:** All-trans retinoic acid (ATRA)’s anticancer mechanisms of action against GC cells.

Fonction	Model of Study	Comments
Cell cycle blocking and differentiation initiation	GC cell lines Tumorspheres PDX	Inhibition of the cell cycle progression by p21WAF1/CIP1 induction [89]. Inhibition of cell proliferation by the inhibition of AP1 transcription factor [90,91]. Downregulation of ERK/MAPK pathway [92]. Induction of the expression of GCSCs differentiation markers [19]. Inhibition of GC cells proliferation in combination with Sorafenib and miRNA in encapsulated nanoparticles [88].
Apoptosis initiation	GC cell lines Tumorspheres PDX	Induction of PDCD4 and cleaved caspase 3 apoptosis markers in GC cell lines, tumorspheres and in PDX models [19]. Induction of apoptosis in GC cell lines by the translocation of TR3 orphan receptor in the mitochondria [93]. Initiation of apoptosis in combination with Sorafenib and miRNA in encapsulated nanoparticles [88].
CSC properties inhibition	GC cell lines Tumorspheres PDX	Inhibition of tumorspheres formation and survival [19]. Reduction of tumor growth in mice subcutaneous xenografts models [19,88,96]. Diminution of liver metastasis after intrasplenic xenograft of GC cells [97]. Reduction of tumor growth in mice subcutaneous xenograft models in combination with Sorafenib and miRNA in encapsulated nanoparticles [88]. Downregulation of the expression of the CSC markers and stemness genes [19].

## Abbreviations

13cisRA	13cis retinoic acid
9cisRA	9cis retinoic acid
ADH	Alcool dehydrogenase
ALDH	Aldehyde dehydrogenase
APL	Acute promyelotic leukemia
ATRA	All-trans retinoic acid
ATPR	4-amino-2-trifluoromethyl-phenyl retinate
CD44	Cluster of differentiation 44
CRABP2	Cellular retinoic acid binding protein 2
CRBP	Cellular retinol binding protein
CSC	Cancer stem cell
CYP26	Cytochrome P450 26A1
CYP450	Cytochrome P450
EMT	Epithelial to mesenchymal transition
GC	Gastric cancers
GCSC	Gastric cancer stem cell
MALT	Mucosa associated lymphoid tissue
N-Cor	Nuclear corepressor
PDX	Patient derived xenograft
PML	Promyelotic leukemia
PLZF	Promyelotic leukemia zinc finger
RA	Retinoic acid
R-ALDH	Retinaldehyde dehydrogenase
RAR	Retinoic acid receptor
RARE	Retinoic acid response element
RII	N-4-(hydroxycarbophenyl) retinamide
RXR	Retinoid X receptor
SMRT	Silencing mediator of RA and thyroid hormone receptor
